# Pre-Slaughter Rest Is Effective in Improving the Physiology and Quality of Nile Tilapia Fillets Subjected to In Vivo Transportation at High Densities

**DOI:** 10.3390/foods14132279

**Published:** 2025-06-27

**Authors:** Maria Ildilene da Silva, Valfredo Figueira da Silva, Marcio Douglas Goes, Sara Ugulino Cardoso, Leonardo Aluisio Baumgartner, Maria Luiza Rodrigues de Souza, Claucia Aparecida Honorato, Robie Allan Bombardelli, Elenice Souza dos Reis Goes

**Affiliations:** 1 Postgraduate Program in Animal Science, Faculty of Agricultural Sciences, Federal University of Grande Dourados, Dourados 79825-070, MS, Brazil; ildilene.mis@gmail.com (M.I.d.S.); valfredo4@gmail.com (V.F.d.S.); douggoes@hotmail.com (M.D.G.); clauciahonorato@ufgd.edu.br (C.A.H.); 2 Postgraduate Program in Fisheries Resources and Fisheries Engineering, State University of Western Paraná, Toledo 85903-220, PR, Brazil; saraugulino@gmail.com (S.U.C.); leonardoaluisiobaumgartner@gmail.com (L.A.B.); rabombardelli@gmail.com (R.A.B.); 3 Department of Animal Science, State University of Maringá, Maringá 87020-900, PR, Brazil; mlrsouza@uem.br

**Keywords:** fish welfare, *Oreochromis niloticus*, pre-harvest stress, processing

## Abstract

This study evaluated the impact of transporting Nile tilapia at stocking densities of 250 kg/m^3^ and 500 kg/m^3^ for 1 h, with post-transport resting periods of 0, 2, 4, and 6 h, on biochemical parameters and fillet quality. A 2 × 4 factorial design was employed for the experiment, with 15 repetitions per treatment. The density of 500 kg/m^3^ resulted in a longer time to rigor mortis after 4 h of rest, while shorter rigor times were observed at 0 and 2 h. Fillets taken from fish transported at 250 kg/m^3^ for 4 h exhibited greater intensities of red and yellow color. The highest weight loss during cooking occurred in fish transported at 500 kg/m^3^ without rest. High-density stocking increased the pH of the fillets, reduced color intensity, and increased weight loss and drip loss. Resting periods of 4 and 6 h resulted in firmer fillets with improved water retention. Fish rested for 6 h at 250 kg/m^3^ recovered glycogen and glucose levels, indicating restored homeostasis. In contrast, fish subjected to high-density transport showed impaired metabolic recovery and compromised fillet quality. These results support the use of resting periods to improve fish welfare and product quality in aquaculture systems.

## 1. Introduction

The increasing global demand for food has driven industries to seek alternatives to enhance the quality of their products. In aquaculture, there is a continuous effort to identify the key factors affecting the quality, tenderness, and shelf life of the final product [1]. These attributes are influenced by a range of in vivo and post mortem factors [2,3,4], which significantly impact the physiological and metabolic responses of fish [5,6].

Although live fish transportation is a potential physiological stressor, it is a crucial operation for the processing industry, as live-transported fish are considered fresher and more flavorful [7]. Therefore, fish welfare is directly linked to product quality, and factors such as stress during transportation and stocking densities must be carefully managed to enhance fish quality, as quality standards are aligned with consumer preferences [8].

Among these factors, stocking density during transport plays a particularly critical role. High densities have been associated with reduced water quality, greater oxygen demand, and accumulation of ammonia and CO_2_, which intensify physiological stress and lead to post mortem alterations such as reduced muscle pH, impaired fillet texture, and color changes [9,10]. Furthermore, high-density transport may prolong the recovery period due to persistent respiratory acidosis and systemic imbalance [11,12].

Studies on pre-slaughter fish welfare indicate that stress begins at the moment of capture and intensifies during transportation [13,14]. During this process, numerous fish are lost while attempting to escape during capture, and mortality rates increase, particularly during long-distance transportation [15]. Contributing factors such as oxygen depletion and high stocking density can further exacerbate these losses, leading to elevated plasma glucose, cortisol, and lactate levels as the fish attempt to maintain homeostasis [16].

In fish, transportation stress has detrimental effects on both physiology and meat quality, as demonstrated in Nile tilapia (*Oreochromis niloticus*) [4,6,17], largemouth bass (*Micropterus salmoides*) [18], channel catfish (*Ictalurus punctatus*) [19], surubim (*Pseudoplatystoma* spp.) [3], Chinese farmed sturgeon (*Acipenser schrenckii*) [20], and Atlantic mackerel (*Scomber scombrus*) [21]. Such adverse effects lead to economic losses for the industry, particularly due to reduced meat quality, as fish often lack sufficient time to recover from stress and return to baseline physiological levels [22].

The response to pre-slaughter stress is characterized by increased muscle activity, including intense swimming in an attempt to escape. This effort leads to heightened anaerobic glycolysis, resulting in the accumulation of lactic acid, which lowers muscle pH, accelerates the onset of rigor mortis, and causes the denaturation of myofibrillar proteins [23]. These factors directly impact texture due to protein denaturation, which coincides with drip loss, reducing water retention capacity and altering fillet color [24,25]. These effects negatively impact both industry profitability and consumer satisfaction [19].

Strategies to mitigate stress in fish include the use of anesthetics during transport [26], the implementation of rest periods immediately after transport [3,19], and the optimization of stocking density during the rest period [17]. These practices play a crucial role in the pre-slaughter phase by helping to restore or maintain homeostasis. While stress responses cannot be entirely eliminated, they can be minimized through improved management practices.

The pre-slaughter rest period, in particular, contributes significantly to physiological recovery after transport. Evidence shows that resting allows for reductions in plasma cortisol, glucose, and nitrogenous waste, contributing to more stable post mortem pH, delayed rigor mortis, and better sensory attributes in fillets [12,27]. However, the effectiveness of the resting period is influenced by both its duration and the stocking density adopted during recovery. Lower densities during rest, such as 50 kg/m^3^, have been shown to facilitate better respiratory recovery and acid–base balance compared with higher densities, such as 300 kg/m^3^, which may perpetuate stress conditions [11].

Optimization of stocking density and pre-slaughter rest periods is essential for minimizing the industry losses caused by post-transport stress [16,26]. Therefore, studying pre-slaughter handling practices is crucial, as physiological responses vary among fish species, with each reacting differently to stressors. This study aimed to evaluate the effects of transportation at medium (250 kg/m^3^) and high (500 kg/m^3^) stocking densities, combined with different post-transport rest periods, on the biochemical parameters and fillet quality of Nile tilapia.

## 2. Materials and Methods

### 2.1. Ethics Statement

The experiment was approved by the Ethics Committee on Animal Use (CEUA/UFGD) of the Federal University of Grande Dourados under protocol No. 16/2022.

### 2.2. Experimental Design

The experiment followed a 2 × 4 factorial design to evaluate the impact of transporting Nile tilapia at two stocking densities (250 and 500 kg/m^3^) for 1 h and four post-transport recovery times (0, 2, 4, and 6 h), along with a control group (fish removed from the tank and immediately slaughtered), resulting in a total of nine treatments. For biochemical and rigor mortis analyses, five fish were sampled per treatment, while ten fish per treatment were used for assessments of fillet quality. These replication numbers were applied uniformly across all treatments to ensure consistency in statistical comparisons.

The fish had an average weight of 967.75 ± 13.98 g and were sourced from net–tank rearing in excavated ponds.

### 2.3. Proceedings

Initially, the fish were removed from the net pond using a hoist. After harvesting, they were weighed on a portable scale and placed in two transport boxes at densities of 250 and 500 kg/m^3^. A fiberglass transport box, designed for live fish transport, was used. The box had a 400 L capacity and was equipped with a diffuser and an oxygen cylinder. The box was filled with clean water from an artesian well, with 6 mg/L of sodium chloride added. Ice was used to maintain the water temperature at 21 °C [28]. Temperature and dissolved oxygen levels were monitored throughout transportation using a portable meter. The transport box was placed in a pickup truck and transported for one hour. Following transportation, 15 fish were sampled from the 0 h treatment, while the remaining fish were transferred to 500 L water tanks at a standard density of 100 kg/m^3^. The fish were held in these tanks for different resting periods (2, 4, and 6 h). At each time point, 15 fish were sampled.

In the control treatment, the fish were removed from the net tank using a hoist and were immediately stunned and slaughtered.

All fish were stunned by cranial percussion, followed by slaughter through gill bleeding [29]. They were then placed in ice water until complete exsanguination and cessation of movement, after which they were processed to obtain fillets. For this, the fish were weighed and subjected to scaling, heading, evisceration, skin removal, and manual filleting. After weighing, the fillets were washed in chlorinated water (2 ppm) and packed in Styrofoam trays covered with polyethylene bags. They were then stored in coolers with ice for 24 h until analysis.

Blood was collected from five fish per treatment immediately after stunning. A total of 3 mL of blood was drawn via caudal puncture using disposable syringes. After blood collection, the fish were bled and subsequently processed for muscle sampling for biochemical analysis.

### 2.4. Blood and Muscle Biochemical Parameters

Blood glucose and lactate levels were analyzed. To obtain serum, the blood samples were centrifuged at 12,000× *g* for 3 min. Glucose [30] and lactate concentrations were determined colorimetrically in the serum.

The levels of glycogen, lactate, lipoperoxidation (LPO), and the enzymes catalase, glutathione S-transferase (GST), and superoxide dismutase (SOD), as well as protein carbonylation, were determined in the muscle.

The previously frozen muscle samples were processed by disrupting the cells and homogenizing them in a phosphate-buffered saline solution (pH 7.2). The homogenized samples were then centrifuged at 11,000 rpm at 4 °C for 10 min. After centrifugation, the supernatant was separated and stored at −80 °C for later biochemical analysis. Glycogen was assessed according to Krisman [31], and lactate was measured using the Lactate Pro2 kit (Arkray, Tokyo, Japan).

Protein concentration was determined using the Bradford method [32], with absorbance readings at 595 nm. After protein quantification, the samples were normalized to a concentration of 1 mg/mL for subsequent tests. Lipoperoxidation was analyzed using the FOX method, as described by Jiang et al. [33], with absorbance readings at 570 nm. Catalase activity was measured according to Aebi [34], with absorbance readings at 240 nm. Glutathione S-transferase activity was assessed using the methodology described by Habig et al. [35], with absorbance readings at 340 nm. Superoxide dismutase activity was determined based on the method described by Crouch [36], with absorbance readings at 560 nm. Finally, protein carbonylation was measured using a spectrophotometer at 360 nm, following the method described by Levine et al. [37].

### 2.5. Analysis of Rigor Mortis

Five fish from each treatment were evaluated from the time of slaughter until rigor mortis was fully reached. Evaluations were conducted every 30 min until the fish reached 100% rigor.

The rigor index (RI) was measured according to Bito [38] and calculated using the following Equation (1):

(1) $$RI=\left(\frac{D0-D}{D0}\right)\times 100$$

where D0 = the distance between the base of the caudal fin and the reference point immediately after death, and D = the distance between the base of the caudal fin and the reference point at the selected time intervals.

RI was used to determine the total time (in minutes) required for each fish to reach 100% rigor mortis. This value, referred to as pre-rigor mortis time, was the variable of interest.

### 2.6. Fillet Yield

Using the data from the whole fish and the fillets, the fillet yield was determined using the following Equation (2):

(2) $$Fillet\;yield=\frac{Fillet\;weight}{Fish\;weight}\times 100$$

### 2.7. Fillet Quality

The indicators evaluated 24 h after slaughter were pH, color, water retention capacity, cooking weight loss, and shear force, which were measured on ten fillets per treatment. Additional fillets were frozen for twenty days at −18 °C, and after thawing, drip loss was determined and texture profile analysis (TPA) was conducted. This procedure also used ten fillets per treatment.

#### 2.7.1. Evaluation of pH, Color, and Shear Force

The pH was measured in duplicate per fillet at two points using a portable digital potentiometer (Testo^®^ 205, Sao Paulo, Brazil) with an insertion electrode designed for meat.

Luminosity measurements were taken on the ventral side of the fillet at six different points per sample. The luminosity values (L*) were assessed using a colorimeter at a 90° angle, at room temperature. In this context, L* defines luminosity (L* = 0 for black and L* = 100 for white), with chroma a* representing the red–green component and chroma b* representing the yellow–blue component, according to the CIELAB system.

The shear force of the fillets was measured using a Brookfield CT3 Texture Analyzer texturometer (Brookfield, Middleboro, MA, USA), equipped with an SMS (Stable Micro Systems) shear cell and a Warner-Bratzler blade. The blade had a thickness of 3 mm, a length of 70 mm, and an angle of 60°. Prior to analysis, the fillets were left at room temperature for approximately 1 h and then the fillets were cut into cubes (approximately 1.5 × 1.5 × 1.1 cm). The analysis was conducted in triplicate per fillet, and the shear force was recorded in grams (g).

#### 2.7.2. Moisture Content and Water Loss in Fillets

Moisture content was determined in duplicate using six fillets per treatment, according to the Association of Official Agricultural Chemists (AOAC) methodology [39]. Water holding capacity (WHC) was determined in triplicate per fillet, following the method described by Barbut [40]. To conduct this method, samples of meat weighing 0.5 g were placed between two circular qualitative filter papers (5.5 cm in diameter, 205 µm thick, and 80 g/m^2^ in weight), which were positioned between two square glass plates, each 8 mm thick. Uniform pressure was applied to the set for five minutes using a 10 kg weight. Afterward, the samples were re-weighed, and the difference between the final and initial weights was expressed as a percentage, as shown in Equation (3).

(3) $$WHC\;(\%)=\frac{Pressed\;Weight}{Initial\;weight}\times 100$$

Water loss during cooking was measured according to the method described by Cason et al. [41]. A 70.0 g sample of meat was weighed, placed in plastic bags, and cooked in a water bath until the internal temperature reached 75 to 80 °C, as measured with a digital thermometer. The samples were then cooled to 30 °C and re-weighed. The difference between the initial and final weights was expressed as a percentage, representing the water loss due to cooking, as shown in Equation (4).

(4) $$Cooking\;loss\;(\%)=\frac{Weight\;after\;cooking}{Initial\;weight}\times 100$$

Drip loss was determined according to the method described by Kaaele et al. [42]. To conduct this method, individually frozen fillets in plastic bags were thawed at 4 °C for approximately 24 h, and the liquid remaining in the plastic bag was weighed. Drip loss was calculated based on the initial weight of the fillet after thawing, as shown in Equation (5).

(5) $$Drip\;loss\;(\%)=\frac{Weight\;of\;exuded\;liquid}{Weight\;of\;thawed\;fillet\;}\times 100$$

#### 2.7.3. Texture Profile Analysis (TPA)

The texture profile analysis (TPA) was conducted in triplicate per fillet using standardized cubes (1.5 cm × 1.5 cm × 1.5 cm), according to the method described by Rodrigues et al. [43]. A TA-XT Plus texturometer (Stable Micro Systems Ltd., Surrey, UK) equipped with a 50 kg load cell and a 36 mm cylindrical probe (P/36) was used. The TPA involved two 60% compression cycles with a 5 s interval between compressions. The pre-test, test, and post-test speeds were set at 1, 1, and 5 mm/s, respectively. The hardness, fracturability, elasticity, cohesiveness, chewiness, and resilience parameters were calculated using the Exponent software package, version 6.1.9.1 (Stable Micro Systems, Surrey, UK).

### 2.8. Statistical Analysis

The results of the biochemical and meat quality parameters were subjected to analysis of variance (ANOVA) using the Factorial ANOVA/General Linear Model procedure in STATISTICA 7.1^®^ software (Statsoft Inc., Tulsa, OK, USA), with a 5% significance level. In the case of significant differences (*p* < 0.05), Tukey’s test was applied to verify the differences between the means. All data are expressed as mean ± standard error of the mean.

For each parameter, the tables present both treatment means (stocking density × rest time) and the main effects of density and rest time alone, calculated by averaging across the other factors. The statistical significance of each factor and their interaction is reported in the final rows of each table.

In addition to the factorial ANOVA, a Pearson correlation analysis was performed to explore potential linear associations between physiological, biochemical, and fillet quality parameters. The Pearson correlation coefficient (r) was calculated using the correlation matrix function, considering a significance level of *p* < 0.05. The complete correlation matrix is included in the Supplementary Materials, Table S1.

## 3. Results

### 3.1. Biochemical Parameters

For muscle glycogen levels (Table 1), an interaction effect was observed between stocking density and resting time (*p* < 0.01). When fish were transported at 250 kg/m^3^ and rested for 6 h, glycogen levels were higher. In contrast, transportation at 500 kg/m^3^ followed by rest for 2, 4, or 6 h resulted in lower muscle glycogen levels. Additionally, lower glycogen levels were observed in animals transported at 500 kg/m^3^ compared with those transported at 250 kg/m^3^.

For blood glucose and lactate levels, although no interaction effect between stocking density and rest times was observed (*p* > 0.05), a pronounced effect from rest times was noted (*p* < 0.01), with higher levels observed in fish that did not undergo post-transport rest and a subsequent decrease to minimum levels after 6 h of rest. In contrast, muscle lactate levels remained stable (*p* > 0.05).

For the antioxidant enzymes (Table 2), an interaction effect between stocking density and rest time (*p* < 0.05) was observed for catalase (CAT) and glutathione S-transferase (GST), where transportation at a lower density combined with 6 h of post-transport rest resulted in higher levels of these enzymes. In contrast, no significant effects (*p* > 0.05) were observed for superoxide dismutase (SOD), lipoperoxidation (LPO), and protein carbonylation (PCO) across the treatments (Table 2).

### 3.2. Fillet Quality

There was a significant effect (*p* < 0.05) of the interaction between stocking densities and resting times on pre-rigor mortis time, red intensity (a), yellow intensity (b), and weight loss due to the cooking of the fillets (Table 3 and Table 4). The longest pre-rigor mortis time was observed at a stocking density of 500 kg/m^3^ with 4 h of rest, while the shortest times were observed at the same density with 0 and 2 h of rest. Regarding fillet color, a stocking density of 250 kg/m^3^ with 4 h of rest resulted in fillets with greater red and yellow intensities. The greatest weight loss during cooking occurred at a stocking density of 500 kg/m^3^ with no resting time after transportation.

When evaluating stocking density individually, the highest stocking density (500 kg/m^3^) resulted in fillets with a higher pH, lower red (a) and yellow (b) intensity, greater weight loss during cooking, and increased drip loss (*p* < 0.05) (Table 3 and Table 4).

When post-transport rest times were evaluated separately, it was observed that 4 and 6 h rest times resulted in fish with a longer pre-rigor mortis time, lower luminosity, higher red intensity, and higher shear force. The pH was lower in fish that did not undergo post-transport rest and higher in those that were rested for 4 h. Fish subjected to 6 h of post-transport rest had fillets with higher water retention capacity and lower drip loss (*p* < 0.05) (Table 3 and Table 4).

The experimental treatments did not affect (*p* > 0.05) the fillet yield or moisture content, nor was there any significant effect on the texture profile (Table 5).

## 4. Discussion

High transportation densities increase energy demands in fish, leading to a reduction in glycogen reserves due to stress [18], as glycogen is utilized to maintain homeostasis [44]. Primary stress responses, such as the release of catecholamines (adrenaline, noradrenaline, and dopamine), help maintain adequate oxygen levels in the blood and trigger an increase in cortisol and plasma glucose levels [45]. These physiological responses are commonly observed during pre-slaughter handling of fish [46].

Although the interaction was not significant, a resting period following transportation facilitated the return to baseline glucose levels. Thus, longer resting durations enhanced the efficiency of meeting the energy demands associated with swimming activity, leading to a greater release of glucose into the bloodstream [47]. Analysis of different stocking densities has shown that blood glucose and lactate levels suggest that fish exhibit adaptive plasticity [18].

The depletion of muscle glycogen to meet energy demands has been reported in fish subjected to pre-slaughter handling [44]. Conversely, fish transported at a stocking density of 500 kg/m^3^ may experience greater exertion, resulting in a more rapid depletion of glycogen reserves. Notably, in the 250 kg/m^3^ group, muscle glycogen levels did not follow a linear trend, with a temporary drop observed at 4 h of rest. This variation may reflect transitional metabolic demands during recovery, such as osmoregulatory adjustments or energy used in muscle repair, which could transiently consume glycogen before full resynthesis. At 6 h, the elevated glycogen levels coupled with low glucose and serum lactate suggest the completion of metabolic recovery and re-establishment of homeostasis.

Under pre-slaughter stress conditions, blood oxygen pressure (PO_2_) decreases [11]. In response to this oxygen depletion, glucose degradation via the fermentative pathway increases, leading to elevated serum lactate levels [48] as a mechanism to maintain homeostasis in fish.

The physiological stress induced by handling and transportation continues to affect fish well-being [49]. The increase in adrenaline in response to stress leads to elevated muscle lactate levels [21]. Upon perceiving the stimulus, the hypothalamus is activated, transmitting electrical signals through the spinal cord to stimulate catecholamine (adrenaline) production [45]. This mechanism activates glycogen breakdown to supply energy during stressful situations [50].

The levels of lactate entering the bloodstream are relatively low compared with those retained in the muscles [11]. This retention can result in muscle damage due to the rupture of connective and muscle tissues post mortem, ultimately leading to myosin denaturation [12,26]. Consequently, fish with high muscle glycogen content that experience pre-slaughter stress may produce fillets with a lower pH, attributed to increased glycolytic activity under anaerobic conditions [3].

In addition to assessing the primary stress response in this study, secondary responses were also investigated by evaluating antioxidant activity in the muscle of Nile tilapia. The increase in catalase (CAT) and glutathione S-transferase (GST) activity represents delayed responses (6 h of rest) to the elimination of reactive oxygen species (ROS) produced by fish transported at 500 kg/m^3^. Elevated CAT and GST activity have been reported in fish subjected to low oxygen conditions [18], which are commonly observed in densification stress [27]. This condition suggests that the antioxidant defense system may play a critical role in supporting adaptive mechanisms observed during pre-slaughter stress.

Under normal conditions, the antioxidant defense system of fish prevents the uncontrolled generation of reactive oxygen species (ROS) through the activity of antioxidant enzymes. The combined activity of glutathione S-transferase (GST) and superoxide dismutase (SOD) is recognized as a key mechanism for preventing oxidative damage to muscle tissue [51], with a direct impact on fillet quality [18]. SOD activity was sufficient to minimize the damage caused by stress in fish transported at a stocking density of 500 kg/m^3^, which may explain the lack of responsiveness in GST activity.

There was no change in lipoperoxidation (LPO) activity, indicating that, under the conditions of this experiment, lipid oxidation did not occur. The imbalance leading to oxidative stress results in lipid oxidation, protein carbonyl formation, genetic damage, and ultimately cell death [51]. Lipid peroxides are considered unstable indicators of oxidative stress, undergoing decomposition to produce more complex and reactive compounds. Among these, 4-hydroxy-2-nonenal (HNE) is commonly used as a biomarker for lipid peroxidation [52].

High oxidative carbonylation of protein (OCP) activity leads to undesirable changes in muscle texture [53], primarily due to increased carbonyl levels, which reflect the oxidation of polyunsaturated fatty acids (PUFAs) [54]. This suggests that LPO may act as a precursor to protein carbonylation (PCO), linking lipid damage to protein damage [54,55]. These findings align with the present study, as no changes were observed in LPO and PCO activity, further demonstrating the high plasticity of Nile tilapia.

The transportation process and pre-slaughter rest time may significantly influence the oxidative stress markers in Nile tilapia. Understanding these oxidative stress indicators contributes to a deeper understanding of the physiological responses of fish during transportation [3], as well as their effects on both welfare and fillet quality.

Extending the pre-rigor mortis period is essential in fish processing. When stress and muscle activity before slaughter are minimized, fish processing (such as filleting and packaging) can be carried out before the onset of rigor mortis, resulting in higher yields and reduced damage to the fish flesh [12]. Additionally, filleting during the pre-rigor mortis process allows the fillets to contract freely during the rigor mortis stage, thus preventing the increased tension that can induce myoseptal breakdown, ultimately contributing to improved fillet yield [4].

The high stocking density (500 kg/m^3^), combined with resting times of either 0 or 2 h after transport, accelerated the rigor mortis process in Nile tilapia. These findings suggest that such treatments accelerated the depletion of the animals’ energy reserves. In fact, pre-slaughter stress depletes muscle and liver glycogen reserves, further accelerating the onset of rigor mortis [49]. High consumption of glycogen due to stress, along with the simultaneous removal of lactic acid by the circulatory system of the live animal, leaves the animal without glycogen reserves [3].

In these cases, after death, rigor mortis continues without the production of lactic acid (pH remains high), resulting in rapid pre-rigor and total rigor without a decrease in pH, a phenomenon known as alkaline rigor mortis [56]. This is supported by the elevated muscle pH observed at a stocking density of 500 kg/m^3^, which negatively impacts meat quality, as reflected in changes in coloration and increased drip loss.

Despite this, lower muscle pH is typically associated with animals that have experienced acute pre-slaughter stress. During stressful situations, intense muscle activity aimed at escaping the stressor leads to glycogen consumption in the absence of oxygen, which increases glucose degradation through the anaerobic glycolytic pathway. This pathway generates H+ ions due to an imbalance between the production and degradation of adenosine triphosphate (ATP), with lactic acid as the end product, resulting in a drop in meat pH and consequently accelerating the onset of rigor mortis [23].

In fact, when evaluating rest times alone, the lowest muscle pH was observed at the shortest rest times (0 and 2 h), while the highest final pH was observed at 4 h post-transport rest. It is interesting to note that longer post-transport rest times (4 and 6 h) resulted in fillets of higher quality, as indicated by lower luminosity, higher firmness, greater water retention capacity, and lower drip loss. Therefore, it is noteworthy that two extremes in muscle pH were observed in this study: high pH due to alkaline rigor mortis and low pH due to acute stress, both of which negatively impacted fillet quality.

An abrupt decline in muscle pH generally causes the denaturation of myofibrillar proteins, resulting in texture damage and reduced meat quality [57]. In Nile tilapia, the decline in post mortem pH negatively affects fillet texture by altering protein solubility, increasing proteolysis, and accelerating denaturation [17]. During pre-slaughter handling and slaughter under stress, muscle pH drops rapidly and intensely, leading to the denaturation of myofibrillar and sarcoplasmic proteins [58].

Protein denaturation has a direct impact on water losses in meat, as it reduces the muscle’s water retention capacity, leading to increased exudate losses during storage and cooking. Cooking weight loss is a parameter that negatively affects meat quality, significantly altering its color and texture, while also influencing its nutritional value, as soluble proteins, vitamins, and minerals are present in the eliminated exudate [12].

In addition, the loss of water from intracellular stores leads to an increase in protein–protein interaction and birefringence of the heme pigment, causing chemical changes that affect the meat’s ability to absorb incident light rays. This results in a reduction in the perception of red and, consequently, paleness of the meat [59]. High luminosity is often an indicator of acute pre-slaughter stress, not only in fish [17], but also in poultry [60] and pigs [61]. In the present study, longer post-transport rest times resulted in fillets with lower luminosity, demonstrating that rest periods of 4 to 6 h are effective for recovering from the stress caused by harvesting and transportation.

This is corroborated by other meat quality parameters, such as higher firmness (represented by higher shear force), higher water holding capacity (WHC), and lower drip loss in animals subjected to longer resting times. Water retention properties, including drip loss and WHC, are important parameters in determining fish quality, as they directly influence the functional properties of muscles, such as juiciness and texture [62].

Consumer studies have indicated that for beef and pork, there is a preference for greater tenderness as a decisive factor for purchase, while the preferred quality for fish is a firm texture with a good WHC [2]. Higher WHC directly reflects lower drip loss, which is desirable for the industry as it provides greater net weight and a more attractive appearance for consumers [62].

In alignment with the findings of this study, for surubim (*Pseudoplatystoma* spp.), resting for 4 to 8 h prior to slaughter was effective in re-establishing homeostasis post-transportation, resulting in fillets of higher quality and an extended pre-rigor mortis period [3].

In Nile tilapia, the stress induced by high transportation densities resulted in an accelerated rate of post mortem changes, decreased water retention capacity, and increased cooking loss [4]. Similarly, in the current study, it was noted that both cooking loss and drip loss were higher at elevated densities, confirming the adverse effects of these transportation conditions on fillet quality.

No differences were detected between treatments in the texture profile of the fillets. Since the instrumental technique of texture profile analysis (TPA) focuses on the similarities in the first two bites during chewing [61], the absence of differences in these analyses may be attributed to the methodology employed. Although not statistically significant (*p* = 0.070), the increase in fillet hardness observed in the 500 kg/m^3^ group after 6 h of rest may reflect the improved muscle structure due to reduced stress and restored homeostasis. This trend is consistent with other fillet quality indicators, such as higher water holding capacity, lower drip loss, and extended pre-rigor mortis time under the same conditions.

To complement the interpretation of treatment effects, a Pearson correlation analysis was performed among the biochemical, oxidative, and technological parameters of the fillets (Supplementary Materials, Table S1). Significant associations were identified between WHC and pre-rigor mortis time (r = 0.309, *p* < 0.05), as well as a negative correlation between WHC and luminosity (L*) (r = –0.331, *p* < 0.01), suggesting that muscle recovery improves water retention and reduces fillet luminosity. Shear force was positively correlated with pre-rigor mortis time (r = 0.335, *p* < 0.05), supporting the interpretation that longer recovery leads to firmer fillets. Drip loss showed a negative correlation with shear force (r = −0.385, *p* < 0.05), reinforcing the link between firmness and exudate control. Overall, these findings demonstrate the physiological connections between stress markers and quality attributes in tilapia fillets subjected to different transport and rest conditions.

## 5. Conclusions

The present study demonstrates that implementing a rest period after high-density live transport significantly improves both the physiological condition of Nile tilapia and the technological quality of their fillets. Resting fish for 4 to 6 h, particularly after high-density transport (500 kg/m^3^), led to significant reductions in serum glucose and lactate, restoration of muscle glycogen, and a longer pre-rigor mortis period. These physiological improvements translated into higher water-holding capacity, lower drip loss, and firmer fillets, even under stressful transport conditions. Without rest, fish exhibited faster rigor mortis onset, pH instability, and greater exudate loss, all of which compromise product yield and shelf life. Therefore, incorporating short-term rest protocols (≥4 h) into pre-slaughter handling can enhance animal welfare, product consistency, and economic efficiency in fish farming.

As the short-term rest protocol proposed in this study requires minimal structural changes, it is easily adaptable across production systems. Future studies should explore its microbiological safety, shelf life implications, and cost–benefit analysis, particularly in commercial-scale operations.

## Figures and Tables

**Table 1:** Levels of muscle glycogen, serum glucose, and lactate in the blood and muscle of Nile tilapia transported for one hour at different stocking densities and subjected to post-transport resting.

Stocking Density + Resting Time After Transportation	Muscle Glycogen (g/L)	Glucose (mg dL^−1^)	Lactate Serum (mmol/L)	Muscle Lactate (mmol/L)
250 kg/m^3^ + 0 h	9.08 ± 0.86 ^bcd^	149.57 ± 14.08	143.37 ± 19.63	12.34 ± 2.63
250 kg/m^3^ +2 h	11.84 ± 1.62 ^abc^	155.07 ± 15.23	67.73 ± 16.70	10.75 ± 1.08
250 kg/m^3^ + 4 h	8.39 ± 0.61 ^bcd^	109.01 ± 8.01	37.48 ± 6.50	10.22 ± 0.75
250 kg/m^3^ + 6 h	12.84 ± 2.01^a^	76.95 ± 6.60	22.98 ± 3.32	10.68 ± 0.79
500 kg/m^3^ + 0 h	12.31 ± 1.81 ^ab^	146.60 ± 11.76	137.33 ± 17.21	11.66 ± 1.18
500 kg/m^3^ + 2 h	7.20 ± 0.91 ^d^	130.85 ± 7.59	43.51 ± 10.35	12.59 ± 1.46
500 kg/m^3^ + 4 h	8.19 ± 0.90 ^cd^	95.77 ± 9.00	27.87 ± 8.61	12.53 ± 2.82
500 kg/m^3^ + 6 h	7.09 ± 0.84 ^d^	80.04 ± 6.37	15.88 ± 2.66	11.25 ± 1.17
Control	8.77 ± 2.31	59.75 ± 2.01	16.99 ± 3.36	17.28 ± 6.37
*p* Values	0.005	0.575	0.873	0.807
Stocking density				
250 kg/m^3^	10.41 ± 0.74 ^a^	122.65 ± 9.03	67.90 ± 12.72	11.00 ± 0.72
500 kg/m^3^	8.78 ± 0.75 ^b^	113.31 ± 7.35	56.14 ± 12.09	12.01 ± 0.83
*p* Values	0.049	0.211	0.178	0.396
Post-transport rest time				
0 h	10.70 ± 1.09	148.09 ± 8.66 ^a^	140.35 ± 12.35 ^a^	12.00 ± 1.37
2 h	9.78 ± 1.24	142.96 ± 8.98 ^a^	54.27 ± 9.71 ^b^	11.67 ± 0.91
4 h	8.29 ± 0.52	102.39 ± 6.09 ^b^	32.67 ± 5.33 ^b^	11.38 ± 1.43
6 h	9.65 ± 1.37	78.49 ± 4.36 ^c^	19.43 ± 2.33 ^c^	10.96 ± 0.67
*p* Values	0.283	0.000	0.000	0.935

Averages in the same column followed by different letters are significantly different according to Tukey’s test (*p* < 0.05).

**Table 2:** Levels of catalase (CAT), superoxide dismutase (SOD), glutathione S-transferase (GST), lipoperoxidation (LPO), and protein carbonylation (PCO) in Nile tilapia transported for one hour at different stocking densities and subjected to post-transport resting.

Stocking Density + Resting Time After Transportation		CAT (U/mL)	SOD (U. mg prot^−1^)	GST (μmol.min^−1^.mg prot^−1^)	LPO (nmol.mg prot^−1^)	PCO (μmol.min^−1^.mg prot^−1^)
250 kg/m^3^ + 0 h		1.88 ± 0.25 ^b^	3.18 ± 0.14	0.03 ± 0.00 ^b^	4.68 ± 3.24	8.36 ± 1.38
250 kg/m^3^ +2 h		1.48 ± 0.33 ^b^	4.48 ± 0.92	0.03 ± 0.00 ^b^	2.74 ± 1.07	4.18 ± 1.20
250 kg/m^3^ + 4 h		2.13 ± 0.32 ^b^	1.82 ± 0.70	0.03 ± 0.01 ^b^	2.21 ± 0.35	4.36 ± 0.74
250 kg/m^3^ + 6 h		4.10 ± 1.05 ^a^	2.64 ± 0.91	0.11 ± 0.05 ^a^	2.03 ± 0.45	5.00 ± 1.19
500 kg/m^3^ + 0 h		2.28 ± 0.19 ^b^	2.48 ± 0.48	0.06 ± 0.02 ^b^	2.61 ± 0.14	6.18 ± 1.14
500 kg/m^3^ + 2 h		2.60 ± 0.39 ^b^	2.99 ± 0.66	0.06 ± 0.02 ^b^	4.48 ± 2.74	5.45 ± 2.09
500 kg/m^3^ + 4 h		2.20 ± 0.16 ^b^	2.71 ± 0.71	0.03 ± 0.01 ^b^	4.49 ± 1.29	5.91 ± 2.75
500 kg/m^3^ + 6 h		2.08 ± 0.12 ^b^	3.18 ± 0.93	0.04 ± 0.00 ^b^	2.22 ± 0.99	5.27 ± 2.10
	Control	3.12 ± 0.01	3.01 ± 1.31	0.04 ± 0.01	3.30 ± 0.10	9.01 ± 1.00
*p* Values		0.009	0.343	0.046	0.601	0.669
Stocking density						
250 kg/m^3^		2.39 ± 0.36	3.03 ± 0.40	0.05 ± 0.01	2.91 ± 0.83	5.48 ± 0.66
500 kg/m^3^		2.29 ± 0.12	2.84 ± 0.33	0.05 ± 0.01	3.45 ± 0.84	5.70 ± 0.94
*p* Values		0.739	0.713	0.854	0.665	0.848
Post-transport rest time						
0 h		2.08 ± 0.16	2.83 ± 0.26	0.05 ± 0.01	3.64 ± 1.70	7.27 ± 0.92
2 h		2.04 ± 0.31	3.73 ± 0.59	0.04 ± 0.01	3.61 ± 1.42	4.82 ± 1.15
4 h		2.16 ± 0.17	2.27 ± 0.49	0.03 ± 0.01	3.35 ± 0.74	5.14 ± 1.29
6 h		3.09 ± 0.60	2.91 ± 0.62	0.07 ± 0.02	2.13 ± 0.51	5.14 ± 1.14
*p* Values		0.077	0.265	0.252	0.797	0.437

Averages in the same column followed by different letters are significantly different according to Tukey’s test (*p* < 0.05).

**Table 3:** Pre-rigor mortis time, fillet yield, pH, colorimetry (L, a, and b*), and shear force of Nile tilapia fillets transported for one hour at different stocking densities and subjected to post-transport resting.

Stocking Density + Resting Time After Transportation	Pre-Rigor Mortis Time (min)	Fillet Yield (%)	pH		Colorimetry		Shear Force (g)
				L *	a *	b *	
250 kg/m^3^ + 0 h	312 ± 22 ^ab^	35.53 ± 0.33	6.40 ± 0.04	43.38 ± 0.43	0.12 ± 0.27 ^b^	−4.38 ± 0.20 ^b^	4621.00 ± 198.39
250 kg/m^3^ +2 h	312 ± 21 ^ab^	35.70 ± 0.63	6.54 ± 0.04	43.47 ± 0.40	0.24 ± 0.17 ^b^	−3.92 ± 0.15 ^ab^	4990.40 ± 288.41
250 kg/m^3^ + 4 h	300 ± 30 ^ab^	35.89 ± 0.46	6.64 ± 0.08	43.51 ± 0.57	1.14 ± 0.26 ^a^	−3.18 ± 0.31 ^a^	5660.00 ± 246.18
250 kg/m^3^ + 6 h	351 ± 28 ^ab^	34.96 ± 0.38	6.57 ± 0.04	42.06 ± 0.35	0.62 ± 0.15 ^ab^	−4.04 ± 0.18 ^ab^	5638.50 ± 286.70
500 kg/m^3^ + 0 h	261 ± 25 ^b^	36.85 ± 0.72	6.63 ± 0.05	43.29 ± 0.66	0.53 ± 0.12 ^ab^	−4.13 ± 0.25 ^ab^	4838.80 ± 201.01
500 kg/m^3^ + 2 h	246 ± 35 ^b^	36.52 ± 0.37	6.70 ± 0.06	42.75 ± 0.37	−0.16 ± 0.21 ^b^	−4.43 ± 0.24 ^b^	4811.50 ± 219.04
500 kg/m^3^ + 4 h	393 ± 30 ^a^	36.02 ± 0.96	6.71 ± 0.05	42.48 ± 0.43	0.03 ± 0.18 ^b^	−4.37 ± 0.28 ^b^	5159.40 ± 226.83
500 kg/m^3^ + 6 h	357 ± 24 ^ab^	35.48 ± 0.60	6.66 ± 0.04	41.74 ± 0.22	0.10 ± 0.11 ^b^	−4.56 ± 0.17 ^b^	5257.10 ± 282.31
Control	360 ± 46	33.15 ± 0.42	6.48 ± 0.04	43.09 ± 0.24	0.52 ± 0.16	−4.56 ± 0.18	5530.90 ± 279.90
*p* Values	0.023	0.785	<0.001	0.728	0.002	0.026	0.488
Stocking density							
250 (kg/m^3^)	319 ± 12	35.52 ± 0.23	6.54 ± 0.03 ^b^	43.11 ± 0.23	0.53 ± 0.12 ^a^	−3.88 ± 0.13 ^a^	5227.44 ± 142.51
500 (kg/m^3^)	314 ± 17	36.22 ± 0.34	6.68 ± 0.02 ^a^	42.56 ± 0.23	0.13 ± 0.09 ^b^	−4.37 ± 0.12 ^b^	5016.73 ± 116.79
*p* Values	0.819	0.100	0.021	0.090	0.004	0.003	0.230
Resting time after transportation							
0 h	287 ± 17 ^b^	36.19 ± 0.41	6.51 ± 0.04 ^b^	43.34 ± 0.38 ^a^	0.32 ± 0.15 ^ab^	−4.25 ± 0.16	4729.90 ± 139.70 ^b^
2 h	279 ± 21 ^b^	36.11 ± 0.37	6.62 ± 0.04 ^ab^	43.11 ± 0.28 ^a^	0.04 ± 0.14 ^b^	−4.18 ± 0.15	4900.91 ± 177.44 ^b^
4 h	347 ± 23 ^ab^	35.95 ± 0.52	6.67 ± 0.05 ^a^	42.99 ± 0.37 ^ab^	0.59 ± 0.20 ^a^	−3.78 ± 0.25	5409.71 ± 172.73 ^a^
6 h	354 ± 18 ^a^	35.22 ± 0.35	6.62 ± 0.03 ^ab^	41.90 ± 0.20 ^b^	0.36 ± 0.11 ^ab^	−4.30 ± 0.13	5447.82 ± 200.64 ^a^
*p* Values	0.011	0.343	0.385	0.010	0.049	0.100	0.008

Data are expressed as mean ± standard error. Means in the same column followed by different letters are significantly different according to Tukey’s test (*p* < 0.05).

**Table 4:** Moisture content, water holding capacity (WHC), cooking weight loss (CWL), and drip loss of Nile tilapia fillets transported for one hour at different stocking densities and subjected to post-transport resting.

Stocking Density + Resting Time After Transportation	Moisture Content (%)	WHC (%)	CWL (%)	Drip Loss (%)
250 kg/m^3^ + 0 h	75.3 ± 0.411	60.77 ± 1.90	13.87 ± 0.94 ^b^	3.90 ± 0.15
250 kg/m^3^ +2 h	75.9 ± 0.551	60.92 ± 1.02	14.81 ± 0.77 ^b^	4.02 ± 0.21
250 kg/m^3^ + 4 h	76.2 ± 0.562	60.33 ± 1.08	17.90 ± 2.09 ^ab^	3.78 ± 0.17
250 kg/m^3^ + 6 h	75.5 ± 0.199	63.53 ± 1.44	13.96 ± 0.64 ^b^	3.04 ± 0.34
500 kg/m^3^ + 0 h	75.7 ± 0.464	57.71 ± 1.18	20.05 ± 0.87 ^a^	4.14 ± 0.05
500 kg/m^3^ + 2 h	76.2 ± 0.455	59.55 ± 1.32	16.68 ± 1.26 ^ab^	5.05 ± 0.50
500 kg/m^3^ + 4 h	75.6 ± 0.233	61.40 ± 0.79	16.33 ± 0.54 ^ab^	3.65 ± 0.10
500 kg/m^3^ + 6 h	75.7 ± 0.472	63.56 ± 0.95	15.90 ± 0.51 ^ab^	4.06 ± 0.60
Control	75.2 ± 0.377	61.02 ± 1.09	14.26 ± 0.88	3.89 ± 0.59
*p* Values	0.664	0.150	0.007	0.230
Stocking density				
250 kg/m^3^	75.70 ± 0.22	60.98 ± 0.74	15.14 ± 0.66 ^b^	3.68 ± 0.15 ^b^
500 kg/m^3^	75.80 ± 0.20	60.56 ± 0.62	17.24 ± 0.49 ^a^	4.22 ± 0.22 ^a^
*p* Values	0.844	0.636	0.007	0.032
Post-transport rest time				
0 h	75.50 ± 0.30	59.24 ± 1.15 ^b^	16.96 ± 0.94	4.02 ± 0.08 ^a^
2 h	76.01 ± 0.34	60.24 ± 0.82 ^b^	15.75 ± 0.75	4.53 ± 0.33 ^a^
4 h	75.92 ± 0.30	60.06 ± 0.78 ^ab^	17.12 ± 1.06	3.72 ± 0.09 ^ab^
6 h	75.63 ± 0.25	63.55 ± 0.84 ^a^	14.93 ± 0.46	3.55 ± 0.38 ^b^
*p* Values	0.568	0.006	0.139	0.030

Data are expressed as mean ± standard error. Means in the same column followed by different letters are significantly different according to Tukey’s test (*p* < 0.05).

**Table 5:** Texture profile analysis (TPA) of Nile tilapia fillets transported for one hour at different stocking densities and subjected to post-transport resting.

Stocking Density + Resting Time After Transportation	Hardness (N)	Fracturability (g)	Elasticity	Cohesiveness	Chewiness (Nmm)	Resilience
250 kg/m^3^ + 0 h	7.85 ± 1.70	622.89 ± 234.21	0.37 ± 0.01	0.25 ± 0.01	0.71 ± 0.15	0.067 ± 0.004
250 kg/m^3^ +2 h	10.07 ± 1.60	757.77 ± 205.45	0.39 ± 0.01	0.25 ± 0.01	0.97 ± 0.16	0.072 ± 0.004
250 kg/m^3^ + 4 h	13.82 ± 2.40	823.37 ± 219.72	0.39 ± 0.01	0.24 ± 0.01	1.28 ± 0.21	0.071 ± 0.004
250 kg/m^3^ + 6 h	8.69 ± 2.05	626.12 ± 254.38	0.37 ± 0.01	0.26 ± 0.01	0.82 ± 0.18	0.072 ± 0.006
500 kg/m^3^ + 0 h	8.15 ± 1.18	944.19 ± 320.83	0.40 ± 0.02	0.27 ± 0.01	0.80 ± 0.09	0.077 ± 0.006
500 kg/m^3^ + 2 h	12.01 ± 2.12	648.36 ± 143.62	0.40 ± 0.02	0.27 ± 0.01	1.12 ± 0.13	0.079 ± 0.005
500 kg/m^3^ + 4 h	10.00 ± 1.85	686.69 ± 182.42	0.42 ± 0.01	0.27 ± 0.01	1.09 ± 0.23	0.078 ± 0.004
500 kg/m^3^ + 6 h	17.38 ± 4.14	662.42 ± 292.45	0.37 ± 0.02	0.26 ± 0.02	1.59 ± 0.45	0.077 ± 0.010
Control	9.22 ± 1.70	342.54 ± 190.80	0.34 ± 0.01	0.25 ± 0.01	0.71 ± 0.08	0.069 ± 0.006
*p* Values	0.070	0.764	0.759	0.815	0.203	0.976
Stocking density						
250 kg/m^3^	9.95 ± 1.01	702.50 ± 108.74	0.38 ± 0.01	0.25 ± 0.01	0.93 ± 0.09	0.071 ± 0.002
500 kg/m^3^	11.88 ± 1.40	735.41 ± 117.52	0.40 ± 0.01	0.27 ± 0.01	1.15 ± 0.14	0.078 ± 0.003
*p* Values	0.279	0.870	0.079	0.082	0.202	0.091
Post-transport rest time						
0 h	8.00 ± 0.99	783.54 ± 195.46	0.38 ± 0.01	0.26 ± 0.01	0.76 ± 0.09	0.072 ± 0.004
2 h	11.04 ± 1.30	703.06 ± 120.64	0.39 ± 0.01	0.26 ± 0.01	1.05 ± 0.10	0.076 ± 0.003
4 h	11.74 ± 1.53	748.82 ± 135.34	0.41 ± 0.01	0.25 ± 0.01	1.17 ± 0.15	0.075 ± 0.003
6 h	13.03 ± 2.56	644.27 ± 184.86	0.37 ± 0.01	0.26 ± 0.01	1.21 ± 0.26	0.074 ± 0.006
*p* Values	0.161	0.940	0.092	0.829	0.185	0.943

Data expressed as mean ± standard error.

## Data Availability

The original contributions presented in the study are included in the article; further inquiries can be directed to the corresponding author.

## Supplementary Materials

The following supporting information can be downloaded at: https://www.mdpi.com/article/10.3390/foods14132279/s1, Supplementary Table S1. Pearson correlation matrix of biochemical, oxidative stress, and fillet quality parameters of Nile tilapia.
