# Supplementary material for: Pre-Slaughter Rest Is Effective in Improving the Physiology and Quality of Nile Tilapia Fillets Subjected to In Vivo Transportation at High Densities
Source: Foods. 2025 Jun 27;14(13):2279. doi: 10.3390/foods14132279 (PMC12248842; doi:10.3390/foods14132279)
Supplement: Supplementary file 1 [file foods-14-02279-s001.zip › foods-3711977-supplementary.pdf]

Supplementary Table S1. Pearson correlation matrix of biochemical, oxidative stress, and fillet quality parameters of Nile tilapia. Significant correlations are marked: p < 0.05 (*), *p < 0.01 (**), and p < 0.001 (***)*.

	pH	L*	a*	b*	CWL	Pre-rigor mortis time	WHC	Shear force	Fillet yield	Moisture	Drip loss	CAT	Muscle glycogen	Glucose	GST	Muscle lactate	Lactate Serum	LPO	PCO	SOD	Hardness	Fracturability	Springiness	Cohesiveness	Gumminess	Chewiness	Resilience
pH	—																										
L*	0.117	—																									
a*	-0.137	0.034	—																								
b*	-0.060	0.292 **	0.608 ***	—																							
**CWL**	0.182	-0.132	0.182	0.095	—																						
**Pre-rigor mortis time**	-0.171	-0.057	0.379 *	0.321 *	0.019	—																					
**WHC**	-0.233 *	-0.331 **	0.184	0.045	-0.147	0.309 *	—																				
Shear force	-0.138	-0.073	0.044	0.041	-0.092	0.335 *	0.094	—																			
**Fillet yield**	0.195	-0.126	-0.219 *	0.046	0.094	0.111	-0.119	0.108	—																		
Moisture	0.045	-0.180	0.151	0.115	0.366 **	-0.057	0.141	-0.019	0.018	—																	
drip loss%	0.390 *	-0.073	-0.003	0.039	0.234	0.074	-0.095	-0.385 *	-0.380	-0.013	—																
CAT	-0.098	0.282	-0.256	-0.047	-0.005	-0.164	-0.186	-0.132	0.124	0.043	-0.081	—															
Muscle glycogen	0.019	0.132	-0.107	0.002	-0.027	-0.001	0.033	-0.270	0.269	0.033	-0.080	0.053	—														
**Glucose**	0.141	0.055	-0.106	-0.068	-0.149	0.207	0.123	0.132	-0.004	-0.078	-0.093	-0.241	0.243	—													
GST	0.079	0.088	-0.141	-0.095	-0.013	-0.241	-0.006	0.038	0.205	0.187	-0.219	0.295 *	-0.092	-0.124	—												
**Muscle lactate**	0.051	0.199	0.015	0.216	-0.195	0.046	-0.043	-0.074	0.051	-0.004	0.034	0.687 ***	-0.034	-0.116	0.062	—											
Lactate Serum	0.127	-0.038	-0.216	-0.171	-0.184	0.029	0.169	0.128	-0.424 **	-0.151	0.074	-0.166	0.112	0.610 ***	-0.109	-0.052	—										
LPO	0.252	-0.179	0.085	-0.035	0.106	0.301 *	0.127	0.180	0.158	-0.019	-0.036	0.161	-0.084	-0.054	0.025	0.228	0.067	—									
PCO	-0.171	-0.148	0.090	-0.307 *	-0.064	-0.100	0.260	0.107	-0.222	-0.044	-0.262	0.041	-0.174	0.139	0.003	0.202	0.182	-0.133	—								
SOD	-0.052	-0.096	0.073	-0.147	-0.165	0.096	-0.023	-0.030	0.086	-0.155	-0.229	-0.505 ***	0.239	0.126	-0.113	-0.517 **	-0.000	-0.233	0.089	—							
Hardness	0.227	0.056	0.137	0.136	0.281 *	0.010	0.090	-0.069	0.112	0.166	0.150	-0.134	-0.010	0.018	-0.140	0.007	-0.182	-0.198	-0.085	0.043	—						
Fracturability	0.202	0.157	0.129	0.345 *	0.146	-0.045	0.147	0.160	0.294 *	0.148	-0.179	-0.161	0.117	0.119	-0.032	-0.028	0.033	-0.138	-0.154	0.024	0.301 *	—					
Springiness	0.027	0.011	-0.047	0.014	-0.213	-0.035	-0.164	-0.022	0.089	-0.122	0.099	0.114	0.041	-0.187	0.092	-0.025	-0.355 *	-0.155	-0.198	0.135	-0.142	-0.276 *	—				
Cohesiveness	-0.206	-0.110	-0.054	0.002	-0.079	0.220	0.120	0.251	-0.018	-0.044	-0.164	0.058	-0.143	-0.123	0.111	-0.126	-0.208	-0.117	-0.126	-0.092	-0.235	-0.308 *	0.515 ***	—			
Gumminess	0.192	0.050	0.082	0.242	0.282 *	0.025	0.155	0.285 *	0.254	0.173	-0.091	-0.119	0.061	0.044	-0.076	-0.022	-0.033	-0.138	-0.146	-0.088	0.468 ***	0.812 ***	-0.205	-0.099	—		
Chewiness	0.231	0.024	0.041	0.069	0.149	0.036	0.075	-0.062	0.092	0.152	0.189	-0.061	-0.009	-0.087	-0.078	0.009	-0.296 *	-0.275	-0.168	0.042	0.917 ***	0.129	0.183	0.047	0.351 *	—	
Resilience	-0.166	-0.117	-0.115	-0.045	-0.050	-0.009	0.068	0.109	0.023	0.079	-0.072	0.302 *	-0.042	-0.228	0.203	0.033	-0.266	-0.221	-0.121	-0.151	-0.244	-0.274 *	0.583 ***	0.876 ***	-0.086	0.035	—
